# The Prophylaxis of Puerperal Convulsions

**Published:** 1879-06-20

**Authors:** 


### *THE PROPHYLAXIS OF PUERPERAL CONVULSIONS.*

Prof. E. S. Dunster read a paper before the last meeting of the Michigan State Medical Society in which he has done very excellent service, by giving expression to a definite plan of prophylaxis, not only as it appears to us, of puerperal convulsions, but of the various symptoms which are liable to the puerperal state and by common consent are now quite generally associated with albuminuria. Dr. Dunster briefly reviews the more prominent theories of the cause, as well as the "prodromic signs and symptoms of the affection which are signals warning us of danger." These are headache, disturbances of the special senses, sight and hearing. "Complete loss of sight is rare, except during the period of active convulsions. No matter what the visual disturbance is, no matter whether it be persistent or intermittent, which last is usually the case, interrogate the kidneys." Next he calls attention to "pain or oppression in the epigastrium. This is enumerated by Chaussier as a symptom of great value, though of less frequency than those just mentioned." Finally we have œdema, though it stands at the head of the list in value.

"Turning now to the consideration of the practical question of treatment and restricting ourselves to the preventive treatment alone, no matter what special therapeutic measures we may resort to, it is easy to group them all under certain well defined indications."

1st. *Relieve the Congestion of the Kidneys.*—Dr. Dunster names rest for the kidney as at the head of the list of means for relief. To secure this the intestinal canal and the skin should be made to do vicariously the work of the kidneys. Hydrogogue purges, therefore, and active diaphoretics are the agents to be relied upon. Diuretics are to be used with caution; Vichy water and acetate of potash may be selected, and these supplemented with infusion of digitalis in short courses (not alcoholic preparations of digitalis). The skin, however, should be kept exceptionally active. Hot vapor baths and friction are advised. Dr. D. suggests that doubtless pilocarpine, the active principle of jaborandi, by its wonderful diaphoretic power, would promise admirable results.

2d. *Counteract the Impoverished State of the Blood, Resulting from the Loss of Albumen.*—Nutritious food and fresh air would, of course, be indicated. The milk diet, either absolute or as a prominent article, has proved of great value as a tonic, and very especially the tincture of the chloride of iron, acting doubly by improving the blood and as a diuretic.

3d. *Quiet the Nerves and Digestive Disturbances.*—Bromide of soda and mono-bromide of camphor are the agents proposed.

4th. *But, Failing in All, Induce Premature Labor.*—Dr. Dunster holds that only a small per cent. of albuminuric subjects becomes eclamptic—say that no more than one in ten. So, also, it is to be borne in mind that delivery does not of necessity cure albuminuria or prevent convulsions. For these and other reasons the induction of premature labor, while it is to be entertained, is to be decided upon with some caution; indeed, there is a sentiment growing stronger, as seen in the writings of Barker, McDonald, Playfair and others, only to resort to premature labor after the careful employment of other methods, that is: "Restrict the operation to cases *where other treatments have been thoroughly tried,* but have failed to secure any amelioration of the symptoms."

The excellent paper that we have thus imperfectly condensed concludes with this cheerful view of our present status: "I cannot close this paper without again expressing the belief that medical art now furnishes a certain method of averting in very many, if not the large majority of cases, the dangers consequent upon the albuminuria of pregnancy, and that it should be our constant aim to early recognize the condition, so that the treatment may be applied in season."—*Obstetric Gazette.*

